# Maintenance of species boundaries in a Neotropical radiation of *Begonia*

**DOI:** 10.1111/mec.13355

**Published:** 2015-09-28

**Authors:** Alex D Twyford, Catherine A Kidner, Richard A Ennos

**Affiliations:** * Ashworth Laboratories, Institute of Evolutionary Biology, The University of EdinburghCharlotte Auerbach Road, Edinburgh, EH9 3FL, UK; † Royal Botanic Garden Edinburgh20A Inverleith Row, Edinburgh, EH3 5LR, UK; ‡ Institute of Molecular Plant Sciences, School of Biological Sciences, University of EdinburghEdinburgh, EH9 3JH, UK

**Keywords:** *Begonia*, hybridization, Mexico, reproductive isolation, speciation, speciation continuum

## Abstract

A major goal of evolutionary biology is to determine the mechanisms generating biodiversity. In *Begonia*, one of the largest plant genera (1900+ species), it has been postulated that the high number of endemic species is a by-product of low gene flow among populations, which predisposes the group to speciation. However, this model of divergence requires that reproductive barriers accumulate rapidly among diverging species that overlap in their geographic ranges, otherwise speciation will be opposed by homogenizing gene flow in zones of secondary contact. Here, we test the outcomes of secondary contact in *Begonia* by genotyping multiple sympatric sites with 12 nuclear and seven plastid loci. We show that three sites of secondary contact between *B. heracleifolia* and *B. nelumbiifolia* are highly structured, mostly containing parental genotypes, with few F1 hybrids. A sympatric site between *B. heracleifolia* and *B. sericoneura* contains a higher proportion of F1s, but little evidence of introgression. The lack of later-generation hybrids contrasts with that documented in many other plant taxa, where introgression is extensive. Our results, in conjunction with previous genetic work, show that *Begonia* demonstrate properties making them exceptionally prone to speciation, at multiple stages along the divergence continuum. Not only are populations weakly connected by gene flow, promoting allopatric speciation, but species often show strong reproductive barriers in secondary contact. Whether similar mechanisms contribute to diversification in other large genera remains to be tested.

## Introduction

Species diversity is unevenly distributed among higher-taxonomic groups, with most genera or families being relatively species poor, and only a minority species-rich (Willis 1922). The groups which are exceptionally species-rich have long captured the interest of biologists, as they show an incredible range of morphological variation on a common theme, and represent particularly successful outcomes of the evolutionary process (Frodin 2004). Such groups include the plant genus *Solanum* (potatoes and relatives), the scarab dung beetle genus *Onthophagus*, and the fungal genus *Cortinarius*, all of which contain over 1500 species (Frodin 2004; Emlen *et al*. 2005; Zotti *et al*. 2014). Studies of these groups may give important insights into the evolutionary mechanisms that give rise to species-richness.

**Fig. 1:**
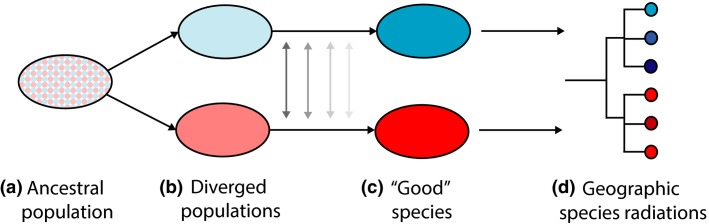
Divergence continuum, from a variable ancestral population (a), through to a large species-rich radiation (d). Figure adapted from Via (2009).

In order for species-rich genera to evolve, an ancestral population must initially diverge, then reproductive barriers must evolve that maintain species distinct identities, prior to further lineage splitting and diversification (Raven *et al*. 2013; Seehausen *et al*. 2014; Figure1). A defining factor at each stage of divergence is the extent of gene exchange among populations and species (Slatkin 1987). In general, the strength of reproductive isolation is positively correlated with genetic distance (Gleason & Ritchie 1998; Price & Bouvier 2002; Le Gac *et al*. 2007; Moyle *et al*. 2007), and as such, gene exchange decreases along the divergence continuum (Mallet *et al*. 2007; Via 2009). At the earliest stages of the divergence continuum (Fig.1B), homogenizing gene flow will prevent the divergence of co-occurring incipient species, unless selection is exceptionally strong (Morjan & Rieseberg 2004), or if loci under divergent selection occur in regions of reduced recombination (Kirkpatrick & Barton 2006; Feder *et al*. 2014; Twyford & Friedman 2015). At the latter stages (Fig.1C), gene flow is expected to be much lower. However, even limited gene exchange among species can have an evolutionary significant impact. Outcomes of interspecific gene flow vary from the extinction of taxa through introgression (Rhymer & Simberloff 1996), to an increased strength of species barriers through reinforcement (Hopkins 2013). To fully understand the process of speciation, it is necessary to understand the role of gene flow both in the origin of species, and in the maintenance of species.

*Begonia* is one of the most species-rich plant genera (>1900 species), and the genus is increasingly used as a model for understanding the evolution of species-rich genera (reviewed in Dewitte *et al*. 2011). Studies along the divergence continuum in *Begonia* have identified many of the processes driving the initial stages of divergence. Populations of African *B. sutherlandii* and *B. dregei*, and American *B. heracleifolia* and *B. nelumbiifolia*, have been shown to be only weakly connected by gene flow and subsequently show strong population substructure (*F*_ST_ range = 0.277–0.937, Matolweni *et al*. 2000; Hughes & Hollingsworth 2008; Twyford *et al*. 2014b). Population substructure is promoted by the characteristic poor pollen and seed dispersal of *Begonia* (Twyford *et al*. 2013b), as well as inbreeding in some species (Ågren & Schemske 1993; Twyford *et al*. 2014b). This population divergence results in the accumulation of geographically restricted genetic incompatibilities (Twyford *et al*. 2014b), and thus, populations of some widespread species are at least part way along the divergence continuum. Consequently, this limited gene exchange may promote allopatric speciation, as demonstrated by the geographically restricted monophyly of clades in phylogenetic analyses (Goodall-Copestake *et al*. 2009; Thomas *et al*. 2011), and the frequent occurrence of narrow endemics (Hughes & Hollingsworth 2008; Figure1D).

While we have a broad understanding of the early stages of the divergence continuum in *Begonia* (Hughes & Hollingsworth 2008), little is known about the maintenance of species in areas of secondary contact (Fig.1C). Studying the outcomes of secondary contact allows us to understand the maintenance of species diversity (Mallet *et al*. 2007), in particular whether species are liable to collapsing through introgression, or whether introgression is resisted and the distinct genetic integrities of species are maintained. Clearly in the case of *Begonia*, even though the propensity for allopatric divergence may be common, this may be of limited evolutionary consequence if species are then recurrently subsumed via hybridization. This could be the case if widespread colonizing *Begonia* species hybridize with narrow endemics, as would be expected if the weak crossing barriers seen in cultivated *Begonia* (Tebbitt 2005; Dewitte *et al*. 2011), translate to weak reproductive barriers in the field. In contrast to this expectation of widespread introgression, our previous crossing experiments have shown that reproductive barriers are present between highly divergent populations of *B. heracleifolia* (Twyford *et al*. 2014b), likely isolated for a relatively short period of time. As such, if reproductive barriers can accumulate rapidly within species, they are even more likely to be present between species.

In this study, we look to understand the maintenance of species in the large Mexican radiation of *Begonia*. To do this, we investigate the genetic structure of zones of secondary contact between two pairs of *Begonia* species that naturally hybridize in Mexico (Burt-Utley 1985). We test between our two contrasting predictions on the maintenance of species: (a) sites of secondary contact represent hybrid zones with rampant introgression, reflecting the weak crossing barriers used to create horticultural *Begonia* hybrids, or (b) secondary contact sites show little introgression, in line with our expectation of rapidly accumulating reproductive barriers observed within species. To distinguish between these scenarios, we estimate the frequency of hybrids, and the generation of each hybrid plant, in multiple sites of secondary contact, using nuclear markers. We also infer the directionality of crossing and potential plastid capture using plastid microsatellites. Overall, these results are used to form a bridge between previous population genetic studies, and species-level phylogenetic work, to shed light on the evolutionary mechanisms responsible for generating and maintaining species diversity in one of the world's largest plant genera (Frodin 2004).

## Material and method

### Study species

**Fig. 2:**
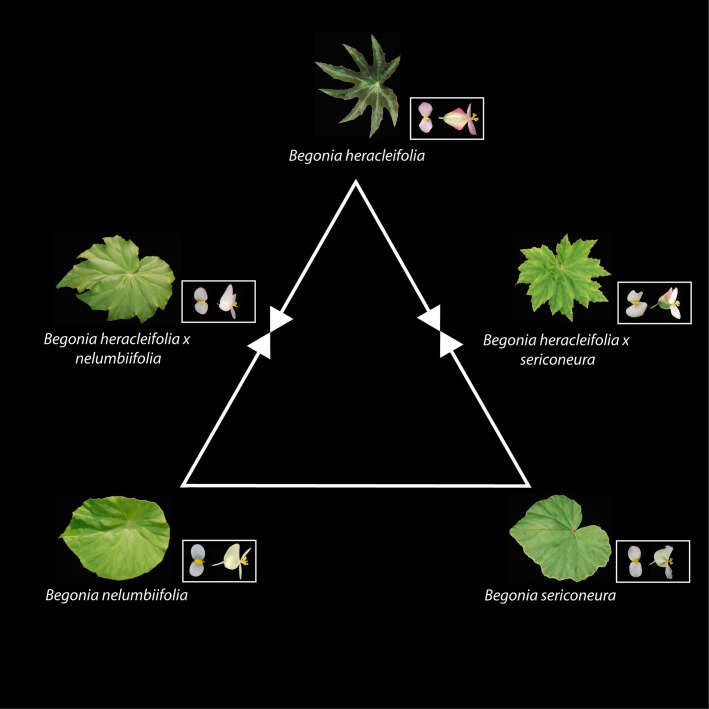
*Begonia* hybrid triangle. Parental leaf shape and staminate and pistillate flowers are shown in each corner, with F1 hybrids between them. Photographs were taken of plants grown in a common greenhouse condition. Natural hybrids between *B. nelumbiifolia* and *B. sericoneura* have not been recorded.

We selected two pairs of species that hybridize in the wild: *B. heracleifolia* and *B. nelumbiifolia*, and *B. heracleifolia* and *B. sericoneura* (Fig.2; Burt-Utley 1985; R. Morris, pers. comm.). These species are best known for their use as foliage begonias in horticulture (e.g. the cultivar *B. heracleifolia* ‘Wisley’). Like other *Begonia*, these three species are thought to be generalist pollinated (Ågren & Schemske 1991) and have been shown to be diploids (Twyford 2012). All three species are close relatives within section *Gireoudia*, one of three sections that together form a major clade of Central American *Begonia* (total 117 species, clade age 9.48 Ma [5.82–13.55 Ma, 95% highest posterior density, HPD], Moonlight *et al*. 2015). This clade is part of the large radiation of Neotropical *Begonia* (>600 species, clade age 12.46 Ma [7.87–17.24 Ma HPD] Moonlight *et al*. 2015).

*Begonia nelumbiifolia* and *B. heracleifolia* are ruderal species that co-occur in disturbed roadside sites, but within sites they are usually separated into different microhabitats (Hoover 1979). *Begonia nelumbiifolia* grows in moist shaded areas, while *B. heracleifolia* has a preference for dry or seasonally dry habitats (Hoover 1979; Burt-Utley 1985). Natural hybrids have been reported at a number of locations (Burt-Utley 1985), and here, we analyse three sites [sites 1, 2 and 3 (S1, S2 and S3), Table 1] in the South of Mexico. Each of these sites are well established roadside populations characterized by mixed secondary vegetation: site S1 was a forest clearing with overgrown grasses and small shrubs, site S2 a roadside cliff with early successional vegetation, and site S3 a roadside bank in shaded woodland. At all three sites, parent individuals were present in their hundreds, with hybrids similarly abundant in S3. In S1 and S2, hybrids were limited to a few plants (discussed below).

**Table 1:** Collection sites of *Begonia* used for this study. Number genotyped refers to *B. heracleifolia* (H), *B. nelumbiifolia* (N), B sericoneura (S), putative hybrids based on morphology (HZ)

					Number genotyped			
Site number	Hybridizing species	Locality details	Latitude	Longitude	H	N	S	HZ
S1	*B. heracleifolia* × *B. nelumbiifolia*	9 km SE of San Andrés Tuxtlas, Veracruz	18.520090	−95.161760	29	29	0	3
S2	*B. heracleifolia* × *B. nelumbiifolia*	Highway 175 between Jasaa and San Juan Bautista, Oaxaca	17.743560	−96.328028	30	40	0	1
S3	*B. heracleifolia* × *B. nelumbiifolia*	San Jeronimo Zoochina, Oaxaca	17.221170	−95.235472	29	30	0	2
S4	*B. heracleifolia* × *B. sericoneura*	Motzorongo, Veracruz	18.669530	−96.787139	41	0	28	32

*Begonia sericoneura* and *B. heracleifolia* are most often found in dry or seasonally dry habitats near roadsides, as well as in open areas of tropical forests (Burt-Utley 1985). One sympatric site was analysed [site 4 (S4), Table 1]. This hybrid zone was large, occurring over at least 100 m of rocky roadside margin, with many hundreds of parent plants and putative hybrids. This site did not show evidence of recent disturbance.

All three species, and their hybrids, are morphologically distinct. *B. nelumbiifolia* possesses green peltate leaves, which are unlobed. In contrast, *B. heracleifolia* produces dark, often anthocyanin-rich (darkly coloured) leaves, which are not peltate, and are deeply lobed. These species also differ in their inflorescence morphology, with *B. nelumbiifolia* producing many small white flowers on large, rounded symmetrical inflorescences, and *B. heracleifolia* a smaller number of larger pink flowers on large upright asymmetric inflorescences. Artificial F1 hybrids are morphologically intermediate and can still be clearly identified from their parents (Fig.2). *B. sericoneura* can be distinguished from *B. heracleifolia* from a suite of traits, including a different leaf shape, leaf colour and leaf hair type (Burt-Utley 1985).

### Sampling strategy and genotyping

For each site, except S1 where access was limited, we estimated the number of each parental species and their putative hybrids, based on morphology. Each species, and early generation hybrids (F1s and F1BCs), can easily be recognized based on leaf shape, particularly leaf serrations, the number of lobes, and whether or not the leaf is peltate (Twyford 2012). We then sampled *c*.30 of each putative parent, and up to 30 putative hybrids, for genetic analysis (Table 1). Our aim was to genotype individuals representative of the full range of intermediate morphological variation that may be present between species, at a given site. Leaf and flower bud material for each individual was collected in silica, prior to DNA extraction with the DNeasy Plant Mini Kit. A total of 101 individuals were analysed at nuclear loci in the sympatric site between *B. heracleifolia* and *B. sericoneura*, and 193 across the three sympatric sites between *B. heracleifolia* and *B. nelumbiifolia*. The reduced number of *B. heracleifolia* × *B. nelumbiifolia* hybrids genotyped reflected the limited number found at each site, and the poor access to site S1. Of the 294 individuals analysed, 84 individuals were newly genotyped for this study and added to the 210 generated in Twyford *et al*. (2013b). Plants were genotyped with the 14 nuclear microsatellite loci described in Twyford *et al*. (2013a) and scored in the same manner. These markers show substantial allele frequency differences between *B. heracleifolia* and *B. nelumbiifolia* (between species *F*_ST_ = 0.47, Twyford *et al*. 2014b), and thus should be highly informative for the genetic analysis of hybridization.

Maternal plastid inheritance has been found in cytological observations (Corriveau & Coleman 1988) and plastid sequencing of experimental crosses (Peng & Chiang 2000) in other *Begonia* species. Therefore, plastid genotyping was used to detect the direction of hybridization. A total of 93 individuals were analysed at plastid loci in the site of secondary contact between *B. heracleifolia* and *B. sericoneura*: 35 *B. heracleifolia*, 28 *B. sericoneura*, and 30 putative hybrids. Fifty-eight of these genotypes were newly generated for this study and added to the 35 *B. heracleifolia* genotypes in Twyford *et al*. (2013b). A total of 111 plants were analysed at plastid loci from two mixed populations of *B. heracleifolia* and *B. nelumbiifolia*: S1, 33 *B. heracleifolia*, 29 *B. nelumbiifolia*, 3 putative hybrids; S2, 20 *B. heracleifolia*, 25 *B. nelumbiifolia*, 1 putative hybrid, as well as the two hybrids from S3. The six hybrid genotypes were newly generated for this study and added to the 105 generated in Twyford *et al*. (2013b). Plants were genotyped with seven plastid microsatellites that reliably amplified across the study taxa, using the same amplification protocol and method of scoring as Twyford *et al*. (2013a).

### Analyses of hybridization

The genetic structure of nuclear microsatellite data were analysed using three complementary approaches. NewHybrids (Anderson & Thompson 2002) was used to assign individuals to the 6 possible genotypic classes after two generations of crossing: parent A-type, backcross A-type, F1-type, F2-type, backcross B-type and parent B-type. Analyses were performed without reference populations following Vähä & Primmer (2006) and repeated for the mixed populations of *B. heracleifolia* and *B. nelumbiifolia* with a reference panel of 12 individuals from across each species range. We performed 100 000-sweeps after a 10 000-sweep burn-in, and a posterior probability cut-off of *q* > 0.9 was used. In addition, we evaluated four generations of hybridization using the extension of NewHybrids proposed by Milne & Abbott (2008).

Second, the program flock (Duchesne & Turgeon 2009) was used, which allocates individuals to one of the user-defined number of genetic clusters (K) with a log-likelihood score (LLOD score). flock is a non-Bayesian approach which operates well even when admixture is high, and when reference populations are not available (Duchesne & Turgeon 2009, 2012). Each mixed population was analysed without reference populations using default parameters and with K = 2. A plot of LLOD scores was evaluated to see whether there were distinct clusters of parents and putative hybrid classes, or whether there is a continuum of LLOD scores suggesting complete admixture.

Third, Bayesian clustering in baps (Corander *et al*. 2008) was used to measure the contribution of each parental genome to the hybrids. Preliminary runs with ‘clustering of individuals’ followed by ‘admixture based on mixture clustering’, where no a priori information is given about pure individuals, performed poorly (results not shown). Therefore, pure individuals were first detected in NewHybrids (*q*_NHZ_ > 0.9), and then defined in the input file for ‘admixture based on predefined populations’, as recommended in the BAPS manual. Default options were selected, except that 10 000 iterations and 5000 reference individuals were used.

We used basic population genetic statistics to look for the sharing of species-specific alleles, and changes in heterozygosity, associated with hybridization. Species-specific (‘private’) alleles were defined as those found in individuals assigned to one of the pure parental categories by NewHybrids, but absent in pure individuals of the other hybridizing species. These were scored by eye. We also tested whether early generation hybrids identified by NewHybrids showed elevated expected heterozygosity, as would be expected in hybrids between divergent parents. This calculation was made using fstat (Goudet 1995).

Plastid haplotypes were defined as the unique combination of alleles at all plastid loci and were defined by eye. The frequency of plastid haplotypes were compared between the putative hybrids and the parents at each sampling site.

### Simulated zones of secondary contact

To assess the discriminatory power of the nuclear markers, and the number of assignment errors made by NewHybrids, we simulated hybrids from the allele frequencies of the nuclear microsatellites of pure individuals at each site (i.e. individuals where *q*_NHZ_ > 0.9). This approach allows simulated and empirical data sets to be compared directly, but assumes the parents have not been introgressed by other species (Burgarella *et al*. 2009). This assumption is supported by their distinct morphologies and the large number of species-specific nuclear alleles and plastid haplotypes (see Results). However, if introgression had occurred, this would simply reduce the power of detecting hybrids (Burgarella *et al*. 2009).

Hybrid swarms were simulated separately for the two species pairs. The parental genotype data for S1 (29 *B. heracleifolia* and 28 *B. nelumbiifolia* individuals), and S4 (39 *B. heracleifolia* and 26 *B. sericoneura* individuals) were used as parents for the two simulated hybrid swarms. Random mating between the parents was simulated by drawing alleles at random from the observed parental allele frequencies using hybridlab v1.0 (Nielsen *et al*. 2006). A total of 90 hybrids (30 F1s and 30 BC to each parent) were simulated for each hybrid swarm, and these were analysed in NewHybrids considering the potential genotypic classes after two and four generations of hybridization, as before. To match the conditions of the empirical data set, we analysed the mixed population of *B. heracleifolia* and *B. sericoneura* without reference populations, while hybridization between *B. heracleifolia* and *B. nelumbiifolia* was analysed with, and without, reference populations (as before). The likelihood of assignment to a genetic cluster (*q*_NHZ_) was averaged across each hybrid class and standard errors calculated, and the number of incorrectly assigned genotypes recorded. As four generations of hybridization generates 210 potential genotypic classes, we followed the approach of Milne & Abbott (2008) to test whether hybrids could confidently be assigned to a single genotypic class, or were assigned to a compound category (e.g. either BC A-type or parent A-type).

## Results

### Genetic diversity of marker loci

The 14 nuclear loci amplified successfully in both species and their hybrids. Two loci were excluded for each sympatric site due to uneven amplification or unexpected repeat motif lengths. For the mixed populations of *B. heracleifolia* and *B. nelumbiifolia*, loci BI6534 and BC332 were excluded, while loci BI5347 and BI3348 were excluded for the mixed population of *B. heracleifolia* and *B. sericoneura*. Repeating analyses on the 10 loci used across all sites gave highly consistent results with the full data sets (results not shown).

All 12 loci were polymorphic within each mixed population of *B. heracleifolia* and *B. nelumbiifolia*, while 11 of the 12 loci were polymorphic in the mixed population of *B. heracleifolia* and *B. sericoneura*, with locus BE332 being monomorphic in S4. The marker loci showed moderate genetic diversity. The mean number of alleles across loci for the mixed population of *B. heracleifolia* and *B. sericoneura* was 4.6 (range 3–7), and the total across all three mixed populations of *B. heracleifolia* and *B. nelumbiifolia* sites was 5.3 (range 2–8).

### Genetic structure in mixed populations of *B. heracleifolia* and *B. nelumbiifolia*

Our analyses revealed all three *B. heracleifolia* × *B. nelumbiifolia* contact zones had a similar genetic structure, in terms of the number of hybrid genotypes present, and the hybrid category of these admixed genotypes (e.g. F1, BC1, etc.). In terms of the frequency of hybrids, all populations were dominated by parental plants, with a minority of hybrid genotypes. In S1, a total of three individuals were assigned by NewHybrids as nonparental genotypes, out of a total of 61 genotyped individuals. In S2 and S3, we identified just 2 and 1 hybrid plants, respectively. All plants identified as parents and hybrids based on morphology, matched their hybrid classification based on genotype (results not shown). In S2 and S3, all individuals identified as putative hybrids from morphology were genotyped, allowing us to crudely estimate the frequency of hybrids. Each parental genotype was present in their hundreds, giving a hybrid frequency of <1% per sampling site.

**Fig. 3:**
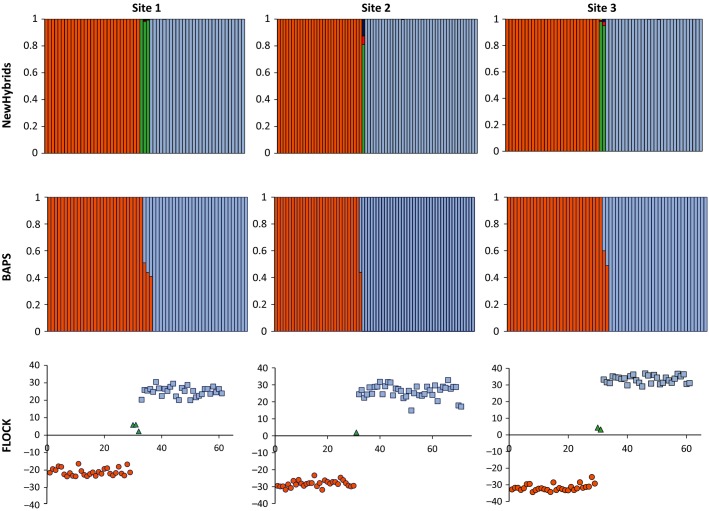
Genetic composition and classification of hybrids in three sympatric populations of *B. heracleifolia* and *B. nelumbiifolia*. NewHybrids analysis (top row): orange, *B. heracleifolia*; blue, *B. nelumbiifolia*; green, F1 hybrid; red, F2; yellow, BC *B. heracleifolia*; dark blue, BC *B. nelumbiifolia*. BAPS (centre row): orange, *B. heracleifolia*; and blue, *B. nelumbiifolia*. FLOCK (bottom row) coloured by most likely class in NewHybrids: orange circles, *B. heracleifolia*; blue squares, *B. nelumbiifolia*; and green triangles, F1 hybrids.

All three analytical methods (BAPS, NewHybrids and flock) support assignment of hybrid genotypes as F1s, with no evidence for the presence of later-generation backcrosses in any mixed population (Fig.3). NewHybrids allocated each of 6 hybrid individuals as F1s (mean *q*_NHZ_ with reference populations = 0.986; without = 0.953), with these genotypes according exactly to their classification based on morphology in the field. The mean contribution of each parents’ genome to the 6 hybrids (estimated in BAPS) was 0.48:0.52 (*B. heracleifolia*:*B. nelumbiifolia*), and in each case, flock identified clusters of *B. heracleifolia* (mean LLOD = −27.06 ± 0.52SE, *n* = 88) and *B. nelumbiifolia* (mean LLOD = 27.98 ± 0.48SE, *n* = 99), with putative hybrids intermediate (mean LLOD = 3.91 ± 0.73SE, *n* = 6). The absence of introgression was further supported by the large number of fixed differences between the hybridizing species. Seventeen of 37 alleles (46%) were found in the pure parental category of *B. heracleifolia* but were absent in the *B. nelumbiifolia* parental individuals. Similarly, 25 of 48 alleles (52%) present in the *B. nelumbiifolia* parents were absent from *B. heracleifolia*. The F1 hybrids combined the fixed allelic differences of the parents and showed an elevated expected heterozygosity (0.63) relative to *B. heracleifolia* (0.26) and *B. nelumbiifolia* (0.37).

In the three mixed populations of *B. heracleifolia* and *B. nelumbiifolia*, a single plastid type was found in *B. nelumbiifolia*, and a total of nine in *B. heracleifolia*. No haplotypes were shared between species. All putative F1 hybrids had the plastid type of *B. nelumbiifolia*.

### Genetic structure in mixed populations of *B. heracleifolia* and *B. sericoneura*

**Fig. 4:**
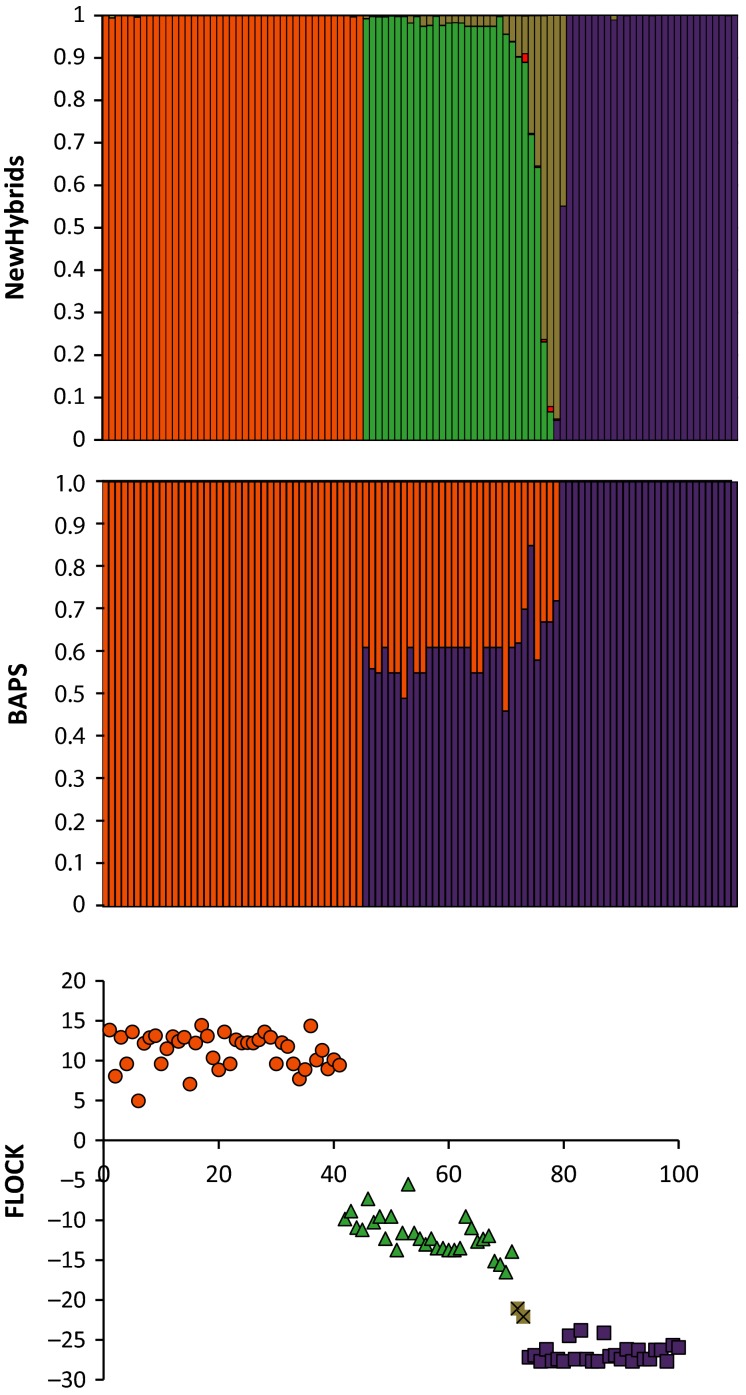
Genetic composition and classification of hybrids in sympatric site 4 (*B. heracleifolia* x *B. sericoneura*). NewHybrids analysis with 6 hybrid categories (top row): orange, *B. heracleifolia*; purple, *B. sericoneura*, green, F1 hybrid; red, F2; yellow, BC *B. heracleifolia*; dark blue, BC *B. sericoneura*. BAPS (centre row): orange, *B. heracleifolia;* and purple, *B. sericoneura*. flock (bottom row) coloured by most likely class in NewHybrids: orange circles, *B. heracleifolia*; green triangles, F1 hybrids; grey crossed squares, putative backcrosses to *B. sericoneura*; purple squares, *B. sericoneura*.

In contrast to the mixed populations of *B. heracleifolia* and *B. nelumbiifolia*, our analysis of hybridization between *B. heracleifolia* and *B. sericoneura* revealed a much greater proportion of F1s, and the presence of backcross genotypes. NewHybrids assigned 27 individuals as pure *B. sericoneura* (mean *q*_NHZ_ = 0.999), 41 pure *B. heracleifolia* (mean *q*_NHZ_ = 0.999), 25 F1s (mean *q*_NHZ_ = 0.980) and 2 BCs to *B. sericoneura* (*q*_NHZ_ = 0.935; Fig.4). Five individuals were not placed in a single category using a stringency of *q*_NHZ_ > 0.9. When stringency was lowered, these were classified as: 3 F1s (*q*_NHZ_ = 0.75), 1 BC to *B. sericoneura* (*q*_NHZ_ = 0.76) and 1 pure *B. sericoneura* (*q*_NHZ_ = 0.55). There was no evidence for any backcrosses to *B. heracleifolia* or F2s. Results considering four generations of crossing following Milne & Abbott (2008) were largely consistent with two generations of crossing (results not shown).

The flock analysis generally supported the NewHybrids results (Fig.4). Three main clusters corresponded to *B. heracleifolia* (mean LLOD = 11.28 ± 0.36SE, *n* = 41), F1s (mean LLOD = −11.90 ± 0.44SE, *n* = 28) and *B. sericoneura* (mean LLOD = −26.73 ± 0.22SE, *n* = 28). Two individuals placed between the F1s and *B. sericoneura* are likely to be BCs (mean LLOD = −21.59 ± 0.52SE). The BAPS analysis grouped 28 individuals as likely pure *B. sericoneura*, 41 as pure *B. heracleifolia* and 31 individuals as admixed (average *q*_BAPS_-value across admixed individuals = 0.40 ± 0.01). Five of these admixed individuals (average *q*_BAPS_ = 0.279, range 0.19–0.33) are more consistent with being backcrosses than F1s. Limited unidirectional introgression was further supported by patterns of fixed allelic differences between the parental classes, with the *B. heracleifolia* parents showing a higher number of unique alleles (23/42, 54%) than *B. sericoneura* (6/24, 25%). Individuals assigned as hybrid genotypes by NewHybrids showed elevated expected heterozygosity (0.419), relative to *B. heracleifolia* (0.280) and *B. sericoneura* (0.114), as would be expected for hybrids between divergent parental progenitors.

The plastid microsatellites revealed three haplotypes in *B. heracleifolia* and one in *B. sericoneura* in S4. No haplotypes were shared between species. The 30 putative hybrids all had the plastid haplotype of *B. sericoneura*.

#### Simulated hybrid swarms

Our simulated hybrid swarms revealed that the genetic diversity of our marker loci is more than sufficient for the accurate assignment of early generation hybrids. Most individuals were correctly assigned when two generations of crossing were considered between *B. heracleifolia* and *B. nelumbiifolia* (>90% assignment success, Table S1, Supporting information), with few individuals not assigned to a hybrid category. Increasing the number of hybrid classes to the 45 unique categories after four generations of crosses (Milne & Abbott 2008) marginally increased the assignment error, and many individuals were assigned to pooled categories.

In the simulated *B. heracleifolia* × *B. sericoneura* hybrid swarm, 143 plants (91.7%) were correctly assigned, and the other 13 (8.3%) were not confidently assigned to a single category. No individuals were miss-assigned. Assignment was also high when all 45 potential hybrid classes after 6 generations of crossing were considered: 140 plants (89.7%) were correctly assigned, and the others were assigned to compound categories (nine plants, 5.8%), remained unassigned (4, 2.6%) or were assigned to the wrong category (3, 1.9%).

## Discussion

There is increasing interest in studying the speciation continuum (Seehausen *et al*. 2014), which follows divergence from the earliest stages of divergence with gene flow (Feder *et al*. 2012; Martin *et al*. 2013), to the evolution of strong reproductive barriers between species (Mallet *et al*. 2007). Yet, there are few study systems that have used this framework to assess the extent of gene exchange at different stages of divergence. Here, we build on previous population-level work in *Begonia* (Matolweni *et al*. 2000; Hughes & Hollingsworth 2008; Twyford *et al*. 2014b), by studying the extent of gene flow between species in sites of secondary contact. In contact zones between *B. heracleifolia* and *B. nelumbiifolia*, hybrids were limited to the F1 generation, and two of the mixed populations were dominated by parental genotypes. Secondary contact sites between *B. heracleifolia* and *B. sericoneura* were largely composed of parental genotypes and F1s, although there was evidence of uncommon unidirectional introgression. Here, we discuss our results in terms of the link between the evolution of reproductive isolation and the origin and maintenance of diversity in large genera such as *Begonia*. Overall, our results suggest that species in the Mexican *Begonia* radiation maintain their distinct genetic identities in sites of secondary contact and that the evolution of strong reproductive barriers may play a role in the maintenance of diversity in a large species radiation.

### Outcomes of natural hybridization in *Begonia*

Our genetic analyses enable us to distinguish between two contrasting scenarios of secondary contact in *Begonia*. We tested whether sites of secondary contact fit a model of rampant gene flow, as may be expected from the weak crossing barriers found in horticultural *Begonia* (Dewitte *et al*. 2011), or one of limited interspecific gene flow, as would be expected if reproductive barriers accumulate rapidly, as seen within *Begonia* species (Twyford *et al*. 2014a). In the mixed populations of *B. heracleifolia* and *B. nelumbiifolia*, backcross hybrids were absent, while in mixed populations of *B. heracleifolia* and *B. sericoneura*, backcrosses were rare. Moreover, plastid haplotypes perfectly tracked species boundaries, even though organellar genomes are particularly prone to being captured by introgression (Gompert *et al*. 2008; Twyford & Ennos 2012). The scarcity of backcross genotypes was the case even though our polymorphic loci would be able to distinguish (at least) first-generation backcrosses from parental and F1 genotypes, if they were present. Our finding of relatively strong reproductive barriers is consistent with other studies, which have generally shown that secondary contact zones in *Begonia* are limited to infertile F1s, with no evidence of backcrosses (e.g. *B.* × *buimontana*, Peng & Chen 1991; *B.* × *breviscarpa*, Peng *et al*. 2010; *B.* x *chungii*, Peng & Shin-Ming 2009; *B.* × *taipeiensis*, Peng & Sue 1991).

The strength of reproductive isolation inferred between the *Begonia* species studied here contrasts with many other diploid species, where widespread introgression is common (e.g. *Aquilegia*, Hodges & Arnold 1994; *Helianthus*, Scascitelli *et al*. 2010; Louisianan *Iris*, Arnold *et al*. 2010; *Populus*, Lexer *et al*. 2005; *Silene*, Minder *et al*. 2007). Strong reproductive isolation was particularly the case for *B. heracleifolia* and *B. nelumbiifolia*, where hybridization was limited to the F1 generation. Moreover, these mixed populations were composed of <1% F1 hybrid individuals, which is much lower than the 25% F1s we would expect if there were equal numbers of parental individuals practicing a selfing rate of 0.5 but outcrossing at random with respect to taxon, assuming no reduction in F1 viability. Our results add *B. heracleifolia* × *B. nelumbiifolia* to the limited list of taxa where hybridization is restricted to the F1 generation (e.g. some *Rhododendron*, Milne *et al*. 2003; Milne & Abbott 2008; *Quercus*, Nason *et al*. 1992; *Costus*, Surget-Groba & Kay 2013; boobys, Taylor *et al*. 2012). The relative paucity of such studies compared with those reporting rampant gene flow may to a large extent be caused by publication bias, as researchers are unlikely to study hybridization in areas where hybrids are thought to be rare. However, studying the frequency of hybrids in highly structured populations, such as those studied here, can be highly informative for understanding the maintenance of species barriers.

While strong reproductive isolation may be a common feature in *Begonia*, we cannot exclude that limited introgression may have an important evolutionary role. Hybridization and introgression may occur between species isolated by strong sterility barriers (Yatabe *et al*. 2007), particularly in hybrid swarms that are long-lived and stable. Long-lived zones of sympatry may be one route to accumulate a sufficient number of F1 genotypes to pass through the sterility bottleneck, and produce backcross hybrids. This seems to be the case in the zone of sympatry between *B. heracleifolia* and *B. sericoneura*, where backcrosses do form despite F1 hybrid sterility. The other documented case of introgression in *Begonia* is between the closely related South East Asian species *B. decora* and *B. venusta*, where a range of intermediate morphologies and admixed AFLP profiles are present in a naturally mixed population (Teo and Kiew, 1999; Kiew *et al*. 2003). The potential for introgression in *Begonia* is also supported by incongruence of phylogenies constructed with different marker loci (Goodall-Copestake *et al*. 2010). More thorough genomic sequencing may be an option to identify introgressed loci (Twyford & Ennos 2012).

### Reproductive barriers involved in species maintenance

Reproductive isolation in plants typically involves a suite of prezygotic and postzygotic barriers (Widmer *et al*. 2009). Our analysis of two hybridizing *Begonia* species, in addition to previous studies, allows us to identify general barriers involved in the maintenance of species barriers in the genus. One important reproductive barrier evident in most studies of natural hybridization in *Begonia* is hybrid sterility. In this study, we see little evidence of introgression, between species where we have previously demonstrated reduced F1 hybrid pollen stainability (<5%) and seed set (3%) relative to a typical interpopulation cross (Twyford 2012). Similar hybrid sterility that may maintain species integrity has been documented in a number of *Begonia* hybrids from Taiwan (Peng & Chen 1991; Peng & Sue 1991; Peng & Shin-Ming 2009; Peng *et al*. 2010). Reduced hybrid pollen fertility is a by-product of irregular hybrid meiosis, due to either genic incompatibilities, chromosomal rearrangements or parental ploidy differences – all of which may play a role in species isolation in *Begonia* (discussed below).

An additional barrier that likely contributes to reproductive isolation is mating system differences. The species studied here all demonstrate moderate levels of inbreeding, with inferred equilibrium selfing rates of 0.40 for *B. heracleifolia* and 0.62 for *B. nelumbiifolia* (Twyford *et al*. 2014b), while the inferred selfing rate for *B. sericoneura* calculated from the newly generated genotypic data presented here is 0.59. Within species, this selfing likely contributes to the strong population substructure (Hamrick & Godt 1996). Between species, differences in selfing rate likely affects the directionality of introgression, as well as the abundance of hybrids (Ruhsam *et al*. 2011). In terms of the direction of introgression, we expect the more highly outcrossing species, here *B. heracleifolia*, to be the pollen donor. This is supported by all hybrids having the plastid type of *B. nelumbiifolia* or *B. sericoneura* (i.e. none with the *B. heracleifolia* plastid type). However, the relatively subtle differences in outcrossing rate between the parents are unlikely to wholly explain the direction of crossing, and other factors, such as flowering time differences or nuclear-cytoplasmic incompatibilities, may also play a role. In terms of the frequency of hybrids, self-fertilization decreases the number of cross-matings (both conspecific and heterospecific) and thus will reduce the potential number of F1s. While we cannot directly quantify the contribution of mating system to reproductive isolation, it could be an important barrier, as recognized in a number of other plant groups (Martin & Willis, 2007; Ruhsam *et al*. 2011; Palma-Silva *et al*. 2015).

The dynamic genome of *Begonia* may also play an important role in reproductive isolation and speciation, with chromosomal restructuring promoting divergence at multiple stages of the divergence continuum. Genome restructuring (inferred from variable population C-values) is associated with reduced stainability of artificial crosses between populations of *B. heracleifolia* (Twyford *et al*. 2014b). These large population differences in genome size could be caused by the accumulation of transposons or large-structural variations in isolated populations via genetic drift (Lande 1985), or selection either on new or existing chromosomal variants (Kirkpatrick & Barton 2006). In turn, these accumulated chromosomal elements could directly contribute to isolation via hybrid sterility, or indirectly, such as by chromosomal inversions sheltering suites of adaptive loci from recombination (Twyford & Friedman 2015). In other members of this radiation, we have mapped a single moderate-effect size pollen sterility QTL to *Begonia* chromosome 4, a chromosome that demonstrates notable differences in marker density between Mexican *Begonia* species (Brennan *et al*. 2012; Twyford *et al*. 2014a). Given that there a number of inverted regions in the *Begonia* map and that polyploidy is common, we anticipate future studies linking genome dynamism to speciation in *Begonia*.

### Linking microevolutionary processes to macroevolutionary patterns in *Begonia*

Our main finding – that introgression is limited between related *Begonia* species – adds to our growing understanding of speciation and reproductive isolation in large plant genera. Sanderson & Wojciechowski (1996) proposed that limited intraspecific gene exchange among populations is a major factor promoting diversification in large plant genera. In strongly substructured species, there is potential for divergence through genetic drift, as well as by selection, as locally adapted alleles will not be dispersed among populations by gene flow, and this may give rise to reproductive barriers accruing among populations (Pinheiro *et al*. 2013). The idea of population processes promoting allopatric speciation and the formation of narrow endemics was developed with respect to *Begonia* by Hughes & Hollingsworth (2008), who related the observed pattern of exceptionally strong genetic substructure within species, to the pattern of geographically restricted monophyly in phylogenies. Thus, they proposed a common mechanism of limited gene exchange promoting divergence in *Begonia*. Our previous work in *B. heracleifolia* (Twyford *et al*. 2014b) has shown that even widespread *Begonia* species show strong population substructure and that there is evidence for the early evolution of reproductive isolation (reduced F1 hybrid pollen viability and seed set) between isolated populations within species. Here, our observation of limited introgression in sites of secondary contact gives further support for this model of speciation. It would seem likely that the rapid accumulation of reproductive barriers seen in isolated *Begonia* populations gives rise to strongly isolated species. These species do not collapse when they co-occur in sites of secondary contact. As such, *Begonia* represents one of the few systems where studies have followed the mechanisms driving diversification from the earliest stages of divergence among populations, through to the origin of large radiations of species that show strong reproductive isolating barriers (Via 2009).

## Data accessibility

Raw microsatellite genotypes and simulated hybrid swarm data: DRYAD http://dx.doi.org/10.5061/dryad.hc6bv.

## Supporting information

**Table S1** NewHybrids assignment for the *B. heracleifolia* x *B. nelumbiifolia* hybrid swarm with different settings.

**Appendix S1** Collection data for the 294 *Begonia* individuals from 4 zones of secondary contact genotyped for microsatellite loci in this study.
