# Supplementary material for: Maintenance of species boundaries in a Neotropical radiation of Begonia
Source: Mol Ecol. 2015 Sep 28;24(19):4982–93. doi: 10.1111/mec.13355 (PMC4600226; doi:10.1111/mec.13355)
Supplement: Supplementary file 1 — Table S1 NewHybrids assignment for the B. heracleifolia x B. nelumbiifolia hybrid swarm with different settings. [file mec0024-4982-sd1.docx]

Table S1 - NewHybrids assignment for the *B. heracleifolia* x *B. nelumbiifolia* hybrid swarm with different settings. Those individuals which were ambiguous in their assignment (e.g. BC2 or BC1) were placed in a compound category. † MCMC analysis consistently ended prematurely.

| **Number of hybrid categories used** | **Reference populations** | **Assigned to correct category** | **Assigned to a compound category** | **Unassigned** | **Incorrect category** |
| --- | --- | --- | --- | --- | --- |
|  |  |  |  |  |  |
| 6 | Yes | 141  (95.3%) | n/a | 6  (4.1%) | 1  (0.75) |
| 6 | No | 143  (96.6%) | n/a | 5  (3.4%) | 0 |
| 45 | Yes | 104  (70.3%) | 34  (23.0%) | 6  (4.1%) | 4  (2.7%) |
| 45† | No |  |  |  |  |

**References**

Nielsen, E. E., Bach, L. A., Kotlicki, P. 2006. HYBRIDLAB (version 1.0): a program for generating simulated hybrids from population samples. Mol. Ecol. Notes, 6: 971-973.
